# Pharmacogenomics of Multiple Sclerosis: A Systematic Review

**DOI:** 10.3389/fneur.2019.00134

**Published:** 2019-02-26

**Authors:** Keli Hočevar, Smiljana Ristić, Borut Peterlin

**Affiliations:** ^1^Clinical Institute of Medical Genetics, University Medical Centre Ljubljana, Ljubljana, Slovenia; ^2^Department of Biology and Medical Genetics, School of Medicine, University of Rijeka, Rijeka, Croatia

**Keywords:** Systematic review, multiple sclerosis, pharmacogenomics, treatment response, personalized treatment

## Abstract

**Background:** Over the past two decades, various novel disease-modifying drugs for multiple sclerosis (MS) have been approved. However, there is high variability in the patient response to the available medications, which is hypothesized to be partly attributed to genetics.

**Objectives:** To conduct a systematic review of the current literature on the pharmacogenomics of MS therapy.

**Methods:** A systematic literature search was conducted using PubMed/MEDLINE database searching for articles investigating a role of genetic variation in response to disease-modifying MS treatments, published in the English language up to October 9th, 2018. PRISMA guidelines for systematic reviews were applied. Studies were included if they investigated response or nonresponse to MS treatment defined as relapse rate, by expanded disability status scale score or based on magnetic resonance imaging. The following data were extracted: first author's last name, year of publication, PMID number, sample size, ethnicity of patients, method, genes, and polymorphisms tested, outcome, significant associations with corresponding *P*-values and confidence intervals, response criteria, and duration of the follow-up period.

**Results:** Overall, 48 articles published up to October 2018, evaluating response to interferon-beta, glatiramer acetate, mitoxantrone, and natalizumab, met our inclusion criteria and were included in this review. Among those, we identified 42 (87.5%) candidate gene studies and 6 (12.5%) genome-wide association studies. Existing pharmacogenomic evidence is mainly based on the results of individual studies, or on results of multiple studies, which often lack consistency. In recent years, hypothesis-free approaches identified novel candidate genes that remain to be validated. Various study designs, including the definition of clinical response, duration of the follow-up period, and methodology as well as moderate sample sizes, likely contributed to discordances between studies. However, some of the significant associations were identified in the same genes, or in the genes involved in the same biological pathways.

**Conclusions:** At the moment, there is no available clinically actionable pharmacogenomic biomarker that would enable more personalized treatment of MS. More large-scale studies with uniform design are needed to identify novel and validate existing pharmacogenomics findings. Furthermore, studies investigating associations between rare variants and treatment response in MS patients, using next-generation sequencing technologies are warranted.

## Introduction

Multiple sclerosis (MS) is a chronic autoimmune disease characterized by the progressive infiltration of inflammatory cells to the central nervous system (CNS), demyelination and axonal damage. Although MS is affecting nearly 2.5 million individuals worldwide (1), its etiology remains largely unexplained. The clinical course of MS is highly heterogeneous, with current evidence suggesting that the combination of environmental and genetic risk factors is involved (1, 2). In general, three types of MS have been characterized, including a relapsing-remitting form of multiple sclerosis (RRMS) (80-85% of MS patients), which might evolve into secondary progressive MS (SPMS), and primary progressive MS (PPMS) manifesting in 15% of patients (3). Also, the response to the existing therapies largely varies between individuals, with estimated non-responder rates ranging up to 50% for interferon beta (IFN-beta) and glatiramer acetate (GA) (4, 5)_._ Although the reasons for that variability remain unclear, several previous studies have implicated the role of genetics in response to MS treatment (6, 7). While RRMS is the main focus of current pharmacogenomic research, as it is the most common and the most responsive to current treatment options, only a few treatments are licensed to slow the progressive form of the disease (3, 8). Approved medicines for MS include immunomodulatory and immunosuppressive drugs and monoclonal antibodies, including subcutaneous and intramuscular interferons, subcutaneous GA, intravenous (iv) natalizumab, oral fingolimod, teriflunomide and dimethyl fumarate, iv mitoxantrone, iv alemtuzumab, and iv ocrelizumab, most of them clinically proven to be effective mainly in reducing annualized relapse rate (ARR) in the early stages of the disease (9). Among most widely prescribed first-line treatments worldwide remain IFN-beta and GA (10), which reduce frequency and severity of relapses in RRMS patients, decrease disease progression rate and improve magnetic resonance imaging outcomes with minimal side effects. Those are the characteristics that are beneficial; however, these drugs are only partially effective, and the response of individual patients to these therapies is highly unpredictable. Current literature suggests that approximately 30-50% of patients do not respond well to first-line therapies (depending on the response criteria used) (5), which is hypothesized to be in part attributed to inter-individual genetic variability. In clinical practice it is often the case that patients should fail to respond to beta-interferons or GA before receiving a second-line treatment (9); moreover, clinical evaluation of response to the therapy requires 1-2 year follow-up (11). It has previously been shown that there is a limited time window for effective intervention, during which the development of early brain atrophy, and thus cognitive and physical deficits, can be minimized more effectively (12). Therefore, the biomarkers that would predict the responsiveness to therapy are indispensable to reduce adverse events and provide the maximized efficacy and safety early in the disease course.

Although pharmacogenomics in clinical practice is increasingly available, there is currently no established genetic or any other clinical biomarker that would reliably predict a response of an individual to selected MS therapy. However, with a growing number of approved treatment options for MS patients in recent years and rapid advances in genomic technologies, personalized medicine has an opportunity to optimize treatment for an individual.

In the present article, we report the results of the conducted systematic review of currently published data on the pharmacogenomics of MS to review the current status of potential pharmacogenomic biomarkers and discuss their future potential in providing the most effective treatment for an individual.

## Methods

The systematic review was conducted according to Preferred Reporting Items for Systematic Reviews and Meta-Analyses (PRISMA) Statement guidelines^1^

### Search Strategy

Articles on the pharmacogenomics of MS therapy published up to October 9^th^, 2018 were searched in the PubMed/MEDLINE database using the combinations of following keywords: multiple sclerosis, pharmacogenomics, pharmacogenetics, therapy response, genome-wide association study (GWAS), genome-wide, gene association study, candidate gene study, polymorphism/s, allele/s, and genetic variants. Search details are given in the Box 1. The search was limited to articles published in the English language. Firstly, articles were screened by title and abstract, next the full content was evaluated for their eligibility. The selection of articles and eligibility evaluation were carried out independently by the first two authors (KH and SR). We discussed discrepancies between authors and reached an agreement on the selection of articles for systematic review. Finally, the main review articles were screened for possible additional publications.

Box 1 **Search details using PubMed database**

((“multiple sclerosis”[All Fields] OR “interferon beta”[All Fields] OR “glatiramer acetate”[All Fields] OR “natalizumab”[All Fields] OR “fingolimod”[All Fields] OR “teriflunomide”[All Fields] OR “dimethyl fumarate”[All Fields] OR “mitoxantrone”[All Fields] OR “alemtuzumab”[All Fields] OR “ocrelizumab”[All Fields]) AND (“pharmacogenomics”[All Fields] OR “pharmacogenetics”[All Fields] OR “pharmacogenetic”[All Fields] OR “pharmacogenomic”[All Fields]))

OR

((“multiple sclerosis”[All Fields] OR “interferon beta”[All Fields] OR “glatiramer acetate”[All Fields] OR “natalizumab”[All Fields] OR “fingolimod”[All Fields] OR “teriflunomide”[All Fields] OR “dimethyl fumarate”[All Fields] OR “mitoxantrone”[All Fields] OR “alemtuzumab”[All Fields] OR “ocrelizumab”[All Fields]) AND (“GWAS”[All Fields] OR “genome-wide”[All Fields] OR “gene association study”[All Fields] OR “polymorphism”[All Fields] OR “polymorphisms”[All Fields] OR “allele”[All Fields] OR "gene variant"[All Fields] OR “alleles”[All Fields]) AND (“treatment response”[All Fields] OR “therapy response”[All Fields] OR “response to therap”[All Fields] OR (response[All Fields] AND ("interferon-beta"[MeSH Terms] OR “interferon-beta”[All Fields] OR ("interferon"[All Fields] AND “beta”[All Fields]) OR “interferon beta”[All Fields])) OR (response[All Fields] AND ("glatiramer acetate"[MeSH Terms] OR (“glatiramer”[All Fields] AND "acetate"[All Fields]) OR "glatiramer acetate"[All Fields])) OR (response[All Fields] AND (“mitoxantrone”[MeSH Terms] OR “mitoxantrone”[All Fields])) OR (response[All Fields] AND ("teriflunomide"[Supplementary Concept] OR “teriflunomide”[All Fields])) OR (response[All Fields] AND (“fingolimod hydrochloride”[MeSH Terms] OR (“fingolimod”[All Fields] AND “hydrochloride”[All Fields]) OR “fingolimod hydrochloride”[All Fields] OR “fingolimod”[All Fields])) OR (response[All Fields] AND (“dimethyl fumarate”[MeSH Terms] OR (“dimethyl”[All Fields] AND “fumarate”[All Fields]) OR “dimethyl fumarate”[All Fields])) OR (response[All Fields] AND (“natalizumab”[MeSH Terms] OR “natalizumab”[All Fields])))).

### Inclusion and Exclusion Criteria

Studies were included if they investigated response or nonresponse to treatment, defined as relapse rate, by expanded disability status scale (EDSS) score or the definition was based on magnetic resonance imaging (MRI), in the association to genetic variability. We included available studies investigating the pharmacogenomics of all currently approved disease-modifying treatment options for MS patients. We excluded articles that: (1) were not written in the English language, (2) were article evaluations, case reports, reviews, study protocols, (3) were using animal model, cell lines, *in silico* studies, (4) investigated response by measuring NAbs/IFN-beta antibodies or studies evaluating therapeutic response by other biochemical tests, (5) were gene expression studies, and (6) investigated adverse drug reactions, such as liver and cardiac injury, acute leukemia and progressive multifocal leukoencephalopathy.

### Data Collection

Two authors (KH and SR) independently extracted the following data from articles: first author's last name, year of publication, PMID number, sample size, ethnic backgrounds of patients, method, genes, and polymorphisms tested, outcome, significant associations with corresponding *P*-values and confidence intervals, response criteria, the duration of the follow-up period and medication investigated. Finally, The Pharmacogenomics Knowledgebase (PharmGKB) was reviewed for possible clinically actionable variants in MS treatments and to search for the level of evidence of the existing MS pharmacogenomic biomarkers. Genes with detected significant associations were annotated for Gene Ontology (GO) molecular functions and biological process using the online PANTHER^TM^ tool version 13.1 (13).

## Results

**Figure 1:**
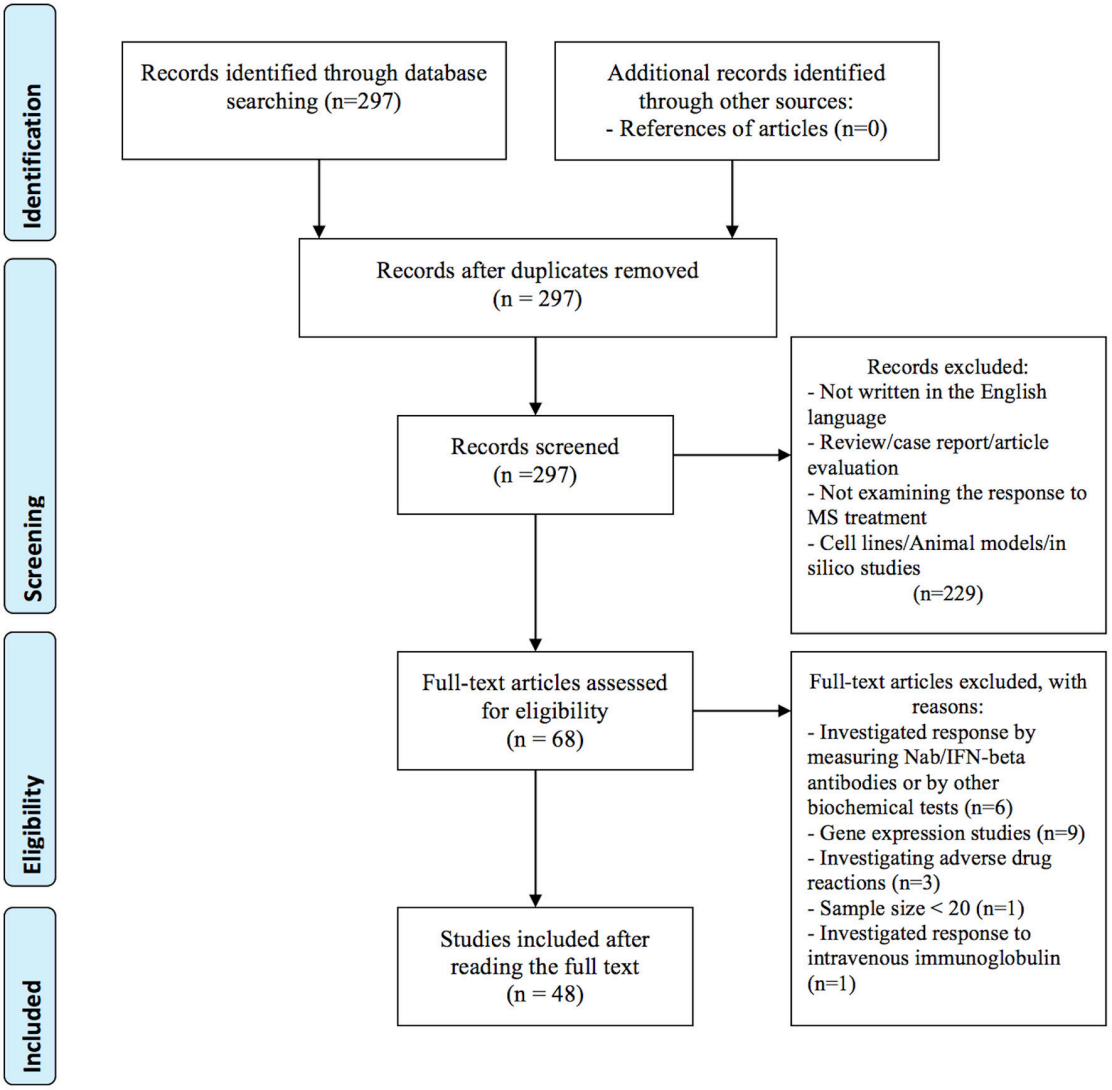
Flow diagram of identification and selection of studies.

In the primary search, we identified a total of 297 articles in the PubMed database. After reviewing titles and abstracts, 229 articles were excluded for the reasons presented in Figure 1. Additional 20 studies were excluded after full-length review, because they investigated treatment response by gene expression (n = 9), by measuring Nab/IFN-beta antibodies or by other biochemical tests (n = 6), investigated adverse drug reactions (n = 3), the sample size was small (less than 20) (n = 1), and investigated response to intravenous immunoglobulin (IVIG) (n = 1). In total, 48 publications investigating the association between genetic variation and treatment response met our inclusion criteria and were included in the systematic review: 40 (83 %) studies investigated treatment response to IFN-beta (5 GWAS and 35 candidate gene studies), 9 (19 %) studies investigated treatment response to GA (one GWAS and 8 candidate gene studies). Among them, four studies investigated the response to both medications; IFN-beta and GA. In addition, we identified two candidate gene studies investigating the response to mitoxantrone and one response to natalizumab. No studies on the pharmacogenomics of newest classes of agents, such as dimethyl fumarate, teriflunomide or fingolimod were identified. Eleven variants with the level of evidence 3 and influence on treatment efficacy were found in the PharmGKB database. Results from candidate gene studies were mostly not replicated, and studies were performed in different populations. Furthermore, genes previously assessed in candidate gene studies showed very little overlap with the significant GWAS associations. Nevertheless, few consistent significant findings (*P* < 0.05) were reported in the candidate gene studies.

### IFN-beta

Interferon-beta 1 is one of the most commonly prescribed disease-modifying therapies for patients with MS. Interferons are endogenous regulatory cytokines that bind to specific IFN alpha/beta receptors found on the surface of the cells of the immune system and consequently change the expression of many genes, depending on cell type - the inflammatory cytokine synthesis is inhibited (IL-12, IL-17, IL-23), while the production of anti-inflammatory cytokines (IL-4, IL-10) increases, which provokes differentiation toward a CD4+ T helper cell type phenotype -Th2 immune response (14). Additionally, interferon reduces the expression of matrix metalloproteases, affects the expression of cell adhesion molecules located on the endothelial surface and on the activated T-cell surface, which results in reduced T-cell activation and reduced lymphocyte migration across the blood-brain barrier (BBB). The potential antiviral activity of IFN-beta has also been proposed (15).

### Candidate Gene Studies IFN-beta

We identified 35 studies investigating the association between genetic variability and response to IFN-beta, four of them also investigating the response to GA. The details of the included studies are presented in Supplementary Table 1. The selection of candidate genes in these studies was mainly based on the proposed mechanisms of action of IFN-beta, and in recent years, studies have also been designed to validate the significant results obtained from genome-wide studies. Some examples of candidate genes investigated were: HLA class II genes, *MXA*, genes coding for interferon receptors *IFNAR1, IFNAR2* and other interferon-stimulated response elements (ISREs), interferon gamma *IFNG*, chemokine receptor *CCR5*, genes related to the type I IFN and TLR pathways, genes coding for GABA and glutamate receptors, genes encoding cytokines and their receptors, innate pattern recognition receptors, antigens *CD46* and *CD58, CTLA4, HAPLN1, ACE* and *APOE* gene.

There are a limited number of studies conducted on the same polymorphisms. Furthermore, among those, the results were largely inconsistent. Sixteen (46%) of included IFN-beta candidate gene studies failed to identify any significant association comparing genetic variation between responders to non-responders. Non-significant associations were repeatedly reported within the HLA locus of class I and/or II (six times) (4, 16–20), in *IFNAR1* and *INFAR2* genes (two times) (21, 22), in *APOE* gene (two times) (23, 24), in *IRF5* gene (25, 26), and *NLRP3* gene (27, 28). Other non-significant associations included *MXA* (29), *HAPLN* (30), *IFNL3* (31), *IRF8* (26), and *GPC5* (26) genes. However, some reproducible significant associations between IFN-beta response and genetic variability have also been detected.

Despite the negative association results between polymorphisms located in the promoter region of the *MXA* gene and IFN-beta response reported by Weinstock-Guttman et al. (29), the significant association was repeatedly demonstrated by two independent studies, which together comprised three different SNPs in *MXA* gene, including rs464138 AA (*P* < 0.0001, OR = 6.23 [95% CI, 2.77–14.03]), rs2071430 G allele (*P* = 0.015, OR = 3.4 [95% CI, 1.1-11.4]), and rs17000900 GG (*P* = 0.018, OR = 2.4 [95% CI, 1.1-5.4]) (32, 33). One of those studies, which investigated 100 ISREs-containing genes in association to IFN-beta response heterogeneity, additionally identified significant associations between *IFNAR1* rs55884088 (GT)_n_ repeat (*P* = 0.036), *LMP7* rs2071543 C allele (*P* = 0.002, OR = 6.4 [95% CI, 1.8-24.1]), and *CTSS* rs1136774 C allele (*P* = 0.02, OR = 0.4 [95% CI, 0.2-0.8]) (32). Another SNP located in the third intron of the *IFNAR1* gene was additionally associated with response to IFN-beta in the study of Sriram et al. (21), suggesting a modest association of rs1012334 A allele with relapse-free status (*P* = 0.030, OR = 0.9 [95% CI, 0.2-1.2]). Furthermore, *IFNAR1* rs1012335 G allele was associated with positive IFN-beta treatment response (34) and was additionally, in allelic combinations, suggested as a marker of choice for IFN-beta treatment over GA (6).

Another repeatedly studied variation is a 32-base pair (bp) deletion of the *CCR5* gene (CCR5^*^d, rs333). A significant association between *CCR5* deletion allele and IFN-beta treatment response in MS patients was confirmed by three independent analyses. In the study of Kulakova et al. (6), CCR5^*^d was more frequently found in Russian MS patients with optimal response to IFN-beta and GA non-responders, while CCR5^*^w/w was enriched in IFN-beta non-responders and GA responders. In the related study, allelic combinations of (CCR5^*^d + IFNAR1^*^G + IFNB1^*^T/T) or (CCR5^*^d + IFNAR1^*^G + IFNG^*^T) were proven to be beneficial for IFN-beta treatment efficacy (34). A significant association between CCR5^*^d and IFN-beta treatment response in MS patients was also detected in the Egyptian population by Karam et al. (35) (*P* = 0.01, OR = 3.2 [95%-CI, 1.1–8.8]).

Certain genotypes of *IRF5* gene polymorphisms (rs2004640 TT, *P* = 0.0006, and rs47281420 AA, *P* = 0.0023) were reported to exert a poor pharmacological response to IFN-beta, with more T2 lesions detected (36). In terms of particular polymorphic loci, the finding *IRF5* rs2004640 was replicated in an independent population within the same study (*P* = 0.037). The study of Vandenbroeck et al. (25) identified the trend toward a greater T allele frequency for the variant of *IRF5* rs3807306 polymorphism in responders (*P* = 0.09), whereas no evidence of an association for *IRF5* rs4728142 was detected. Evidence that an AA genotype of *IRF8* rs17445836 polymorphism influences event-free survival in IFN-beta treated subjects was also found (*P* = 0.017, OR = 0.45 [95% CI, 0.2-0.9]) (19). Contrary, the study conducted in a Danish cohort of patients by Sellebjerg et al. (26), failed to identify any association between polymorphisms located in *IRF5* (rs2004640, rs3807306, rs4728142) and in *IRF8* (rs13333054 and rs17445836) genes.

The number of studies attempting to validate or further investigate the results of GWAS studies is limited. Polymorphous loci in the *GPC5* gene were reproducible with candidate-gene study of Cénit et al. (rs10492503 AA, *P* = 0.018, OR = 3.0 [95% CI, 1.3-6.6]; rs1411751 GG, *P* = 0.012, OR = 3.7 [95% CI, 1.5-9.4]), and GWAS by Byun et al. (rs10492503, rs9301789) (37), while the candidate gene study conducted by Sellebjerg et al. (26) yielded non-significant result. The aim of the study conducted by Bustamante et al. (38) was to further investigate the results of two GWAS studies that highlighted the importance of genes playing role in toll-like receptor (TLR) pathways, type I interferon (IFN)-induced genes, and genes coding for GABA and glutamate receptors. An investigation of 384 polymorphisms located in those genes, detected only two significant polymorphisms (rs2277302 in *PELI3* gene, *P* = 0.008, and rs832032 in *GABRR3, P* = 0.006 gene). Overall association of polymorphisms located in these pathways was therefore not confirmed (38).

As the evidence of polygenic nature of IFN-beta treatment response, allelic combinations (*JAK2-IL10RB-GBP1-PIAS1* and *JAK2-IL10-CASP3*) were detected as significant, while no significant association of tested individual polymorphisms was found (39). In another study, MS patients with non-GCC haplotypes (rs1800896, rs1800871, rs1800872) of the *IL10* gene experienced fewer new MRI T1-contrast enhancing lesions than patients with the GCC haplotype (40).

Other positive associations included: intronic polymorphism rs2542109 of the *USP18* gene, *TGFB1* rs1800469, *TRAILR-1* rs20576, *CD46* rs2724385, *GPC5* rs10492503 and rs1411751 polymorphisms, polymorphic microsatellite located in the first intron of the *IFNG* gene, and *CD58* rs12044852 polymorphism.

Significant associations (*P* < 0.05) between treatment response and IFN-beta, identified in at least one study, are presented in Table 1.

**Table 1:** Significant associations from candidate gene studies and IFN-beta MS treatment response along with selected gene ontology (GO) annotations. *P*_*perm*_, *P*-value permutation test; *P*_*f*_, *P*-value exact Fisher's test.

**Gene symbol**	**Full gene name**	**dbSNP ID; *P*-value; OR, 95-CI**	**GO molecular function**	**GO biological processes**
*MXA promoter region* (32), (33)	Myxovirus resistance protein	**rs464138** AA genotype, *P* < 0.0001, OR = 6.23 [2.77–14.03] **rs2071430** G allele, *P* = 0.015, OR = 3.4 [1.1–11.4] **rs17000900** GG genotype, *P* = 0.018, OR = 2.4 [1.1–5.4]	–	–
*ACE* (41)	Angiotensin-converting enzyme	**I/D** D allele, *P* = 0.022, OR = 2.43 [1.13–5.20]	Carboxypeptidase activity Endopeptidase activity Metallopeptidase activity peptidyl-dipeptidase activity	Regulation of vasoconstriction Regulation of blood pressure Neutrophil mediated immunity Antigen processing and presentation of peptide antigen via MHC class I Angiotensin maturation Mononuclear cell proliferation
*CCR5*, (6), (34), (35)	C-C motif chemokine receptor 5	**rs333/** CCR5^*^d *P* = 0.01, OR = 3.2 [1.1–8.8] Another study, *P_*f*_* = 0.036, OR = 1.9 In allelic combinations: CCR5^*^d+IFNAR1^*^G+IFNB1^*^T/T *P_*perm*_* = 0.017, OR = 14.3 [1.7–119.4] CCR5^*^d**+**IFNAR1^*^G**+**IFNG^*^T *P_*perm*_* = 0.035, OR = 2.8 [1.3–6.0] Comparative to GA: CCR5^*^d+IFNAR1^*^G+DRB1^*^15+TGFB1^*^T *P_*f*_* = 0.00054, *P_*perm*_* = 0.004, OR = 13.2 [3.1–55.4] CCR5^*^w/w+CTLA4^*^G *P_*perm*_* = 0.017	Chemokine receptor activity Protein binding C-c chemokine receptor activity	Cellular defense response MAPK cascade Dendritic cell chemotaxis Calcium ion transport Immune response Inflammatory response Chemotaxis
*CD58* (42)	CD58 molecule	**rs12044852** CC genotype, *P* < 0.05	Receptor binding	Immune response Cellular response to interferon-gamma Leukocyte migration
*PELI3* (38)	Pellino E3 ubiquitin protein ligase family member 3	**rs2277302** *P* = 0.008, OR = 1.29 [1.07–1.56]	Protein binding ubiquitin protein ligase activity	Toll signaling pathway Immune response
*GABRR3* (38)	Gamma-aminobutyric acid type A receptor rho3 subunit (gene/pseudogene)	**rs832032** *P* = 0.006, OR = 1.31 [1.08–1.59]	Gaba receptor activity signal transducer activity	Regulation of biological process Response to stimulus
*IFNAR1* (6), (21), (32), (34)	Interferon alpha and beta receptor subunit 1	**rs55884088** (GT)n repeat, *P* = 0.036 **rs1012334** A allele, *P* = 0.030, OR = 0.9 [0.2–1.2] In allelic combinations **rs1012335**: CCR5^*^d+IFNAR1^*^G+IFNB1^*^T/T *P_*perm*_* = 0.017, OR = 14.3 [1.7–119.4] CCR5^*^d+IFNAR1^*^G+IFNG^*^T *P_*perm*_* = 0.035, OR = 2.8 [1.3–6.0] Comparative to GA: CCR5^*^d+IFNAR1^*^G+DRB1^*^15+TGFB1^*^T *P_*f*_* = 0.00054, *P_*perm*_* = 0.004, OR = 13.2 [3.1–55.4]	Cytokine receptor activity protein binding signal transducer activity	Regulation of biological process Response to stimulus Type I interferon signaling pathway Defense response to virus *TGFB1* (6), (34)
*TGFB1* (6), (34)	Transforming growth factor beta 1	**rs1800469** C allele, *P* = 0.042, OR = 9.2 [0.2–70.4] Comparative to GA: CCR5^*^d+IFNAR1^*^G+DRB1^*^15+TGFB1^*^T *P_*f*_* = 0.00054, *P_*perm*_* = 0.004, OR = 13.2 [3.1–55.4]	Transforming growth factor beta receptor binding	MAPK cascade apoptotic process Biosynthetic process Mononuclear cell proliferation Nitrogen compound metabolic process Regulation of phosphate Regulation of transcription from RNA polymerase II promoter Response to endogenous stimulus Transmembrane receptor protein Serine/threonine kinase signaling pathway Positive regulation of regulatory T cell differentiation Leukocyte migration
*USP18* (43)	Ubiquitin specific peptidase 18	**rs2542109** AA genotype, *P* = 0.041, OR = 1.8 [1.0–3.1]	Thiol-dependent ubiquitinyl hydrolase activity thiol-dependent ubiquitin-specific protease activity isg15-specific protease activity protein binding	Protein deubiquitination Ubiquitin-dependent protein catabolic process regulation of type I interferon mediated signaling Pathway regulation of inflammatory response
*TRAILR-1* (44)	TRAIL receptor1	**rs20576** CC genotype, validation cohort: *P* = 8.88 × 10^−4^, *Pc* = 0.048, OR = 0.30 [0.1–0.6]	Cysteine-type peptidase activity Protein binding Signal transducer activity Tumor necrosis factor-activated receptor activity	Apoptotic process Cell proliferation Cytokine-mediated signaling pathway Immune response Regulation of biological process Regulation of catalytic activity Response to biotic stimulus Response to stress Single-multicellular organism process Leukocyte migration
*IFNG* (34), (45)	Interferon gamma	polymorphic microsatellites in the first intron, **(CA)**_**12**_, *P* = 0.013, OR = 0.5 [0.3–0.9] **(CA)**_**13**_, *P* = 0.04, OR = 1.8 [1.0–3.1] **(CA)**_**14**_, *P* = 0.009, OR = NA **(CA)**_**15**_, *P* = 0.005, OR = 0 [0–0.6] In allelic combinations **rs2430561:** CCR5^*^d+IFNAR1^*^G+IFNG^*^T, *P_*perm*_* = 0.035, OR = 2.8 [1.3–6.0]	Cytokine activity	Immune response Cellular response to lipopolysaccharide Defense response to virus
*IFNB1* (34)	Interferon beta 1	In allelic combinations **rs1051922**: CCR5^*^d+IFNAR1^*^G+IFNB1^*^T/T *P_*perm*_* = 0.017, *P_*f*_* = 0.0028, OR = 14.3 [1.7–119.4]	Interferon-alpha/beta receptor binding	JAK-STAT cascade biosynthetic process Cell differentiation Cell proliferation Cytokine-mediated signaling pathway Type I interferon signaling pathway Hemopoiesis Natural killer cell activation Protein phosphorylation Regulation of phosphate metabolic process Response to stress Single-multicellular organism process Defense response to virus Regulation of innate immune response
*IRF5* (36)	Interferon regulatory factor 5	**rs2004640** TT genotype, *P* = 0.0006, *P*_replication_ = 0.037 **rs47281420** AA genotype, *P* = 0.0023	Sequence-specific dna binding transcription factor activity	Regulation of transcription from RNA polymerase II promoter Response to interferon-gamma Defense response to virus
*IRF8* (19)	Interferon regulatory factor 8	**rs17445836** AA genotype, *P* = 0.017, OR = 0.45 [0.2–0.9]	Sequence-specific dna binding transcription factor activity	Regulation of transcription from RNA polymerase II promoter Cellular response to interferon-gamma Cellular response to lipopolysaccharide
*CD46* (46)	CD46 molecule	**rs2724385** TT genotype, *P* = 0.006, OR = 3.5 [1.4–8.9] AT genotype, *P* = 0.007, OR = 0.40 [0.20–0.79]	Endopeptidase activity complement binding virus receptor activity	Positive regulation of regulatory T cell differentiation Blood coagulation Cell-cell adhesion Complement activation signal transduction
*GPC5* (30)	Glypican 5	**rs10492503** AA genotype, *P* = 0.018, OR = 3.0 [1.3–6.6] **rs1411751** GG genotype, *P* = 0.012, OR = 3.7 [1.5–9.4]	Heparan sulfate proteoglycan binding	Glycosaminoglycan metabolic process Glycosaminoglycan catabolic process Glycosaminoglycan biosynthetic process Retinoid metabolic process
*Il10* (39), (40)	Interleukin 10	In allelic combinations, ***JAK2*****-*****IL10*****-*****CASP3*** *P_perm_* = 0.001 non-GCC haplotypes (**rs1800896, rs1800871, rs1800872**) *P* = 0.04	Protein binding cytokine activity	Positive regulation of endothelial cell proliferation Positive regulation of transcription, DNA-templated Negative regulation of B cell proliferation Negative regulation of cytokine secretion involved in immune response Response to molecule of bacterial origin Negative regulation of myeloid dendritic cell activation Cellular response to lipopolysaccharide
*LMP7* (32)	Proteasome subunit beta 8	**rs2071543** C allele, *P* = 0.002, OR = 6.4 [1.8–24.1]	Threonine-type endopeptidase activity	Viral process Type I interferon signaling pathway Transmembrane transport MAPK cascade Protein polyubiquitination Protein deubiquitination
*CTSS* (32)	Cathepsin S	**rs1136774** C allele, *P* = 0.02, OR = 0.4 [0.2–0.8]	Cysteine-type peptidase activity	Toll-like receptor signaling pathway Antigen processing and presentation Adaptive immune response Proteolysis Antigen processing and presentation of exogenous peptide antigen via MHC class II
*HLA-DBR1, HLA-A, HLA-B* (6), (47)	Histocompatibility complex	HLA-DRB1^*^04 allele, *P* = 0.008OR = 1.94 [1.19–3.17] HLA-B^*^15 allele, *P* = 0.03OR = 0.29 [0.10–0.84] Comparative to GA: CCR5^*^d+IFNAR1^*^G+DRB1^*^15+TGFB1^*^T *P_*f*_* = 0.00054, *P_*perm*_* = 0.004, OR = 13.2 [3.1–55.4]	Antigen binding, receptor binding	Antigen processing and presentation Type I interferon signaling pathway Regulation of immune response

### GWAS Studies and IFN-beta

Currently, five GWAS studies investigating an association between IFN-beta treatment response and genetic variation were carried out. GWAS study, which investigated SNPs in HLA- and non-HLA genes in association with the development of antibodies to IFN-beta therapy, was excluded from this review (48). None of the GWAS studies reported similar results, but they uniquely suggested that multiple genes influence the treatment response to IFN-beta. Furthermore, on the level of genes, most of the results were in deviation with previously conducted candidate-gene studies, thus providing novel candidate genes that might be involved in response to IFN-beta treatment. However, it is important to note that some potential candidate genes reported by independent GWAS studies were involved in the same biological pathways.

In the first GWAS study, conducted in 2008 by Byun et al. (37), authors found out that many of the detected differences between responders and non-responders were located in genes involved in ion channels and signal transduction pathways. Additionally, the authors also suggested that genetic variants in heparan sulfate proteoglycan genes (*HAPLN1*) might be useful as possible clinical predictors of response to MS therapy. Results of the second GWAS study conducted by Comabella et al. (11), indicated the importance of the glutamatergic system (*GRIA3* gene) in patients response to IFN-beta therapy. The GWAS study, conducted by Esposito and colleagues followed in 2015 and reported candidate intronic polymorphism rs9828519 in the *SLC9A9* gene encoding for sodium/hydrogen exchanger found in endosomes. For this gene, a broader role in MS pathogenesis, beyond treatment with IFN-beta, was also proposed. The gene product was functionally characterized to inhibit the development of pro-inflammatory CD4+ T cells (7).

In the study of Mahurkar et al. (49), none of the SNPs reached the level of genome-wide significance. The strongest associations were observed for *FHIT* gene and followed by variants in *GAPVD1* and near *ZNF697* gene. In the discovery stage of this study, samples were individually genotyped using Illumina® arrays, which distinguishes it from previously published GWAS studies where pooled genotyping was performed. A recent GWAS study conducted by Clarelli et al. (50) investigated long-term treatment response considering a 4-year follow-up study period and included only patients with extreme phenotypes of treatment responses. In contrast, all of the previous GWAS studies have taken into account 2-year follow up period. In summary, alterations in the genes involved in immunoregulatory processes, the glutamatergic system (*GRIK2* and *GRM3*), and signal transduction (*GAPVD1*) reached the highest significance.

Lack of overlap between GWAS studies likely reflects the differences in definitions of responders or non-responders, furthermore, GWAS studies covered populations of various ethnicities, including Italian, German, Spanish, and Australian. Also, the methodology was based on different genotyping platforms - first two GWAS studies used Affymetrix genotyping platforms, covering 100 000 and 428 867 SNPs, respectively, while Illumina arrays were used in all of the later studies (Illumina® Human 660-Quad platform, Illumina® 2.5M platform, Illumina® OmniExpress BeadChip and Illumina® OMNI-5M array). However, we showed that genes identified by GWAS studies were significantly enriched for ionotropic glutamate receptor signaling pathway (GO:0035235). Top-ranking results from GWAS studies are summarized in Table 2.

**Table 2:** Genes with detected significant associations with response to IFN-beta in at least one study and top-ranking results from GWAS studies along with selected gene ontology (GO) biological processes and molecular functions.

**Gene**	**Gene name**	**dbSNP ID; *P*-value; OR, 95-CI**	**GO molecular function**	**GO biological procesess**
*GRHL3* (50)	Grainyhead-like 3	**rs6691722** *P_*meta*_* = 5,96 × 10^−6^, OR = 0.4221	Sequence-specific DNA binding transcription factor activity	Biosynthetic process Cellular process Nitrogen compound metabolic process Regulation of transcription from RNA polymerase II promoter
*NINJ2* (50)	Nerve injury-induced protein 2	**rs7298096** *P_*meta*_* = 9.8 × 10^−5^, OR = 0.51	Protein binding	Cellular process Nervous system development
*TBXAS1* (50)	Thromboxane A synthase 1	**rs4726460** *P_*meta*_* = 7.41 × 10^−5^, OR = 0.4662	Heme binding monooxygenase activity	Oxidation-reduction process Positive regulation of vasoconstriction
*GRM3* (50)	Glutamate receptor, metabotropic, 3	**rs2237562** *P_*meta*_* = 2.21 × 10^−4^, OR = 0.47	Binding Glutamate receptor activity Signal transducer activity	G-protein coupled receptor signaling pathway Neurological system process Neuron-neuron synaptic transmission Regulation of biological process Response to stimulus
*GRIK2* (50)	Glutamate receptor, ionotropic, kainate 2	**rs1475919** *P_*meta*_* = 1.89 × 10^−4^, OR = 2.37	Ionotropic glutamate receptor activity extracellular-glutamate-gated ion channel activity	Synaptic transmission Glutamatergic glutamate receptor signaling pathway Intracellular protein transport Cellular calcium ion homeostasis Excitatory postsynaptic potential
*GAPVD1* (49)	GTPase-activating protein and vps9 domains 1	**rs10819043** *P_*combined*_*_(discovery&replication)_ = 5.83x10^−5^ **rs10760397** *P_*combined*_* = 6.51 × 10^−5^ **rs2291858** *P_*combined*_* = 1.67 × 10^−4^	Guanyl-nucleotide exchange factor activity Protein binding Small GTPase regulator activity	Endocytosis Intracellular protein transport
*ZNF697* (49)	Zinc finger Protein 697	**rs10494227** *P_*combined*_* = 8.15 × 10^−5^	Molecular function Metal ion binding DNA binding	Biological process Regulation of transcription, DNA-templated Transcription, DNA-templated
*FHIT* (49)	Fragile histidine triad gene	**rs760316** *P_*combined*_* = 6.74 × 10^−6^	Nucleotide phosphatase activity	–
*SLC9A9* (7)	Solute carrier family 9 (sodium/hydrogen exchanger), member 9	**rs9828519** *P_*discovery*_* = 4.43 × 10^−8^ *P_*replication*_* = 7.78 × 10^−4^	Cation transmembrane transporter activity Hydrogen ion transmembrane transporter activity	Cellular process Homeostatic process
*ADAR* (11)	Adenosine deaminase, RNA-specific	**rs2229857** A allele, *P* = 0.02, OR = 2.1 [1.1–4.0]	DNA binding RNA binding Deaminase activity Hydrolase activity Kinase activator activity Protein binding	RNA localization Cell cycle Protein metabolic process Purine nucleobase metabolic process Response to stimulus
*IFNAR2* (11)	Interferon-alpha, -beta, and -omega receptor 2	**rs2248202** C allele, *P* = 0.04, OR = 1.9 [1.0–3.7]	Cytokine receptor activity protein binding signal transducer activity	Regulation of biological process Response to stimulus
*CIT* (11)	Citron rho-interacting serine/threonine kinase	**rs7308076** C allele, *P* = 0.006, OR = 2.4 [1.3–4.4]	Protein kinase activity	Cell cycle Intracellular signal transduction
*ZFAT* (11)	Zinc finger gene in autoimmune thyroid disease 1	**rs733254** G allele, *P* = 0.02, OR = 2.1 [1.1–4.0]	DNA binding transcriptional activator activity, RNA polymerase II core promoter proximal region sequence-specific binding nucleic acid binding	Hematopoietic progenitor cell differentiation Transcription from RNA polymerase II promoter Spongiotrophoblast layer development Positive regulation of transcription from RNA polymerase II promoter
*ZFHX4* (11)	Zinc finger homeobox 4	**rs11787532** C allele, *P* = 0.04, OR = 2.3 [1.0–5.2]	RNA binding structural constituent of cytoskeleton	Cellular component morphogenesis Cellular defense response Ectoderm development Embryo development Gamete generation Immune system process Mesoderm development Muscle organ development Negative regulation of apoptotic process Nervous system development
*STARD13* (11)	Start domain-containing protein 13	**rs9527281** T allele, *P* = 0.02, OR = 2.0 [1.1–3.7]	–	GTPase activator activity Protein binding Lipid binding
*GRIA3* (11)	Glutamate receptor, ionotropic, AMPA 3	**rs12557782** G allele, *P* = 0.002, OR = 2.7 [1.5–5.2]	Binding Glutamate receptor activity Ligand-gated ion channel activity Signal transducer activity	Cell surface receptor signaling pathway Ion transport Regulation of biological process Response to stimulus
*GPC5* (37)	Glypican 5	**rs10492503**, *P_*f*_* = 0.0070, Aa:AA: OR = 0.51 [0.29–0.89]; aa:AA: OR = 0.32 [0.13–0.81] **rs9301789**, *P_*f*_* = 0.0100, aa:AA: OR = 2.83 [1.41–5.66]	Heparan sulfate proteoglycan binding	Glycosaminoglycan metabolic process Glycosaminoglycan catabolic process Glycosaminoglycan biosynthetic process Retinoid metabolic process
*COL25A1* (37)	Collagen, type xxv, alpha-1	**rs794143** *P_*f*_* = 0.0370, aa:AA: OR = 2.27 [1.17–4.40]	Amyloid-beta binding Heparin binding	Collagen catabolic process Axonogenesis involved in innervation
*HAPLN1* (37)	hyaluronan and proteoglycan link protein 1	**rs4466137** *P_*f*_* = 0.0040, Aa:AA: OR = 0.50 [0.29-0.84]; aa:AA: OR = 0.22 [0.06–0.72]	Binding Extracellular matrix structural constituent	Cell adhesion Cellular process Nervous system development Single-multicellular organism process Skeletal system development
*CAST* (37)	Calpastatin	**rs10510779** *P_*f*_* = 0.0420, Aa:AA: OR = 1.95 [1.09–3.48]	Cysteine-type endopeptidase inhibitor activity protein binding	Proteolysis
*NPAS3* (37)	Neuronal PAS domain protein 3	**rs4128599** *P_*f*_* = 0.0240, Aa:AA: OR = 1.85 [1.07–3.17]	Sequence-specific DNA binding RNA polymerase II transcription factor activity	Biosynthetic process Cellular process Nitrogen compound metabolic process
*TAFA1* (37)	Protein FAM19A1	**rs4855469** *P_*f*_* = 0.0100, aa:AA: OR = 3.04 [1.47–6.28]	Protein binding	–
*FLJ32978* (37)	WD repeat-containing protein 64	**rs952084** *P_*f*_* = 0.0050, aa:AA: OR = 0.15 [0.42–0.56]	–	–

### Candidate Gene Studies and Glatiramer Acetate

Glatiramer acetate is another widely prescribed disease-modifying therapy for patients with MS, with a complex and yet not fully understood mechanism of action. GA is a heterogeneous mixture of synthetic polymers made of random sequences of four amino acids (51). It acts through immunomodulatory actions to the cells of innate and acquired immune response. Through binding to MHC Class II molecules, it participates in the generation of GA-specific T-cells and shifts their phenotype from pro-inflammatory helper-T types 1 and 17 (Th1/Th17) to anti-inflammatory regulatory T cells (Tregs) and helper-T type 2 (Th2) cells. Additionally, GA-specific T-cells are able to migrate through the BBB, where they induce local secretion of anti-inflammatory cytokines at the site of the lesions (51).

Eight candidate gene studies investigating an association between polymorphisms and treatment response to GA met our inclusion criteria. Four of them were mentioned above, as they also investigated IFN-beta treatment response. Detailed data on studies is summarized in Supplementary Table 1. Hypothesis-driven approaches have primarily investigated genes involved in the mechanism of action of GA. Contrary to IFN-beta response, the HLA class I /II genes have been repeatedly positively associated with GA treatment response. The HLA DRB1 ^^*^^1501 allele was demonstrated to influence the response to GA therapy in the cohort of 44 Italian RRMS patients (*P* = 0.008) (4) and in the cohort of 332 American patients, HLA-DBR1^^*^^1501/1501 genotype was significantly enriched among GA-responders (rs3135388, *P*_*AG*/*AA*_ = 0.015, OR = 2.7 [95% CI, 1.2-6.0]) (19). The related study conducted in 64 American subjects with RRMS, showed that the presence of HLA DR15 or DQ6 alleles or the absence of DR17 and DQ2 alleles is nominally associated with a favorable clinical response (52). The authors also demonstrated that the presence of the DR15-DQ6 haplotype and the absence of the DR17-DQ2 haplotype is significantly associated with positive treatment response (52). Furthermore, in a cohort of 296 Russian patients, the nominally significant association of HLA-DRB1^^*^^4 allele with a positive response to GA was detected comparing responders to nonresponders and intermediate responders, *P*_*f*_ = 0.015, OR = 2.02 [95% CI, 1.11–3.67] (53).

One of the first pharmacogenetics candidate-gene studies on GA, reported a significant association between GA response and two SNPs, rs71878 in a T-cell receptor beta (*TCRB*) gene (*P* = 0.015, OR = 6.85 [95% CI, 1.45–31.9]) and rs2275235 in the cathepsin S (*CTSS*) (*P* = 0.014, OR = 11.59 [95% CI, 1.6–81.9]) (54). Nominally significant associations were shown for additional genes *MBP, CD86, FAS, IL1R1*, and *IL12RB2*. However, in the same experiment, no significant association for the HLA-DRB1^^*^^1501 allele was identified, suggesting the genetic heterogeneity of this region among the different populations as the possible reason (54).

Using comparative pharmacogenomics approach investigating allelic combinations of *CCR5, IFNAR1, TGFB1, DRB*, and *CTLA4* genes, the CCR5^^*^^w/w genotype was the most enriched in GA responders compared to IFN-beta responders (6). In the most recent study examining association between GWAS identified MS susceptibility loci and efficacy of GA therapy in a Russian population of 296 RRMS patients, five SNPs were associated by themselves with event-free phenotype: *EOMES* rs2371108 T allele, *CLEC16*A rs6498169 A allele, *IL22RA2* rs202573 GG genotype, *PVT1* rs2114358 A allele, and HLA-DBR1^^*^^4 (*P*_*f*_ = 0.032-0.00092). Authors demonstrated increased significance levels when taking into account biallelic and triallelic combinations of these genes with additionally included polymorphic variants of *TYK2, CD6, IL7RA* and *IRF8* genes (53).

Genes with at least one detected significant association (*P* < 0.05), along with GO biological processes and molecular functions, are presented in Table 3.

**Table 3:** Genes with detected significant associations with response to GA from candidate gene studies and GWAS studies along with selected gene ontology (GO) biological processes and molecular functions. *P*_*perm*_, P-value permutation test; *P*_*f*_, *P*-value exact Fisher's test.

**Gene**	**Gene name**	**dbSNP ID, *P*-value; OR, 95-CI**	**GO molecular function**	**GO biological processes**
**CANDIDATE GENE/ LOCUS STUDIES**				
*IFNAR1* (6), (55)	Interferon alpha and beta receptor subunit 1	**rs1012335** in allelic combination DRB1^*^15+TGFB1^*^T+CCR5^*^d+IFNAR1^*^G *P_*perm*_* = 0.0056 DRB1^*^15+CCR5^*^d+IFNAR1^*^G *P_*f*_* = 0.014 DRB1^*^15+TGFB1^*^T+IFNAR1^*^G *P_*f*_* = 0.015 TGFB1^*^T+CCR5^*^d+IFNAR1^*^G *P_*f*_* = 0.025 Comparative to IFN-beta: CCR5^*^d+IFNAR1^*^G+DRB1^*^15+TGFB1^*^T *P_*f*_* = 0.00054, *P_*perm*_* = 0.004, OR = 13.2 [3.1–55.4]	Cytokine receptor activity, protein binding, signal transducer activity	Regulation of biological process, response to stimulus Type I interferon signaling pathway Defense response to virus
*CCR5* (6), (55)	C-C motif chemokine receptor 5)	**rs333** in allelic combination DRB1^*^15+TGFB1^*^T+CCR5^*^d+IFNAR1^*^G *P_*perm*_* = 0.0056 DRB1^*^15 **+**TGFB1^*^T+CCR5^*^d *P_*perm*_* = 0.013 DRB1^*^15+CCR5^*^d+IFNAR1^*^G *P_*f*_* = 0.014 TGFB1^*^T+CCR5^*^d+IFNAR1^*^G *P_*f*_* = 0.025 Comparative to IFN-beta: CCR5^*^d+IFNAR1^*^G+DRB1^*^15+TGFB1^*^T *P_*f*_* = 0.00054, *P_*perm*_* = 0.004, OR = 13.2 [3.1-55.4] CCR5^*^w/w+CTLA4^*^G *P_*perm*_* = 0.017	Chemokine receptor activity Protein binding C-C chemokine receptor activity	Cellular defense response MAPK cascade Dendritic cell chemotaxis Calcium ion transport Immune response Inflammatory response Chemotaxis
*TGFB1* (6), (55)	Transforming growth factor beta 1	**rs1800469** in allelic combination DRB1^*^15+TGFB1^*^T+CCR5^*^d+IFNAR1^*^G *P_*perm*_* = 0.0056 DRB1^*^15+TGFB1^*^T+CCR5^*^d *P_perm_* = 0.013 DRB1^*^15+TGFB1^*^T+IFNAR1^*^G *P_*f*_* = 0.015 TGFB1^*^T+CCR5^*^d+IFNAR1^*^G *P_*f*_* = 0.025 Comparative to IFN-beta: CCR5^*^d+IFNAR1^*^G+DRB1^*^15+TGFB1^*^T *P_*f*_* = 0.00054, *P_*perm*_* = 0.004, OR = 13.2 [3.1–55.4]	Transforming growth factor beta receptor binding	MAPK cascade Apoptotic process Biosynthetic process Cell differentiation Mononuclear cell proliferation Nitrogen compound metabolic process Protein phosphorylation Regulation of phosphate Regulation of transcription from RNA polymerase II promoter Response to endogenous stimulus Transmembrane receptor protein Serine/threonine kinase signaling pathway Positive regulation of regulatory T cell differentiation
*CTLA4* (6)	Cytotoxic T-lymphocyte associated protein 4	**rs231775** Comparative to IFN-beta: CCR5^*^w/w+CTLA4^*^G *P_*perm*_* = 0.017	Cytokine activity	Cellular defense response cellular process
*HLA-DBQ1* (52) *HLA-DBR1* (4), (6), (19), (52), (53), (55)	Histocompatibility complex	HLA-DBR1^*^1501, *P* = 0.008 **rs3135388**, *P*_AG/AA =_ 0.015, OR = 2.7 [1.2-6.0] **HLA-DR17**, *P* = 0.0077 **–DR15**, *P* = 0.0062 **–DQ2**, *P* = 0.0028 **–DQ6**, *P* = 0.0044 Haplotypes **–DR15-DQ6**, *P* = 0.0062 **–DR17-DQ2**, *P* = 0.0077 HLA-DRB1^*^4, *P_*f*_* = 0.015, OR = 2.02 [1.11–3.67] Allelic combinations DRB1^*^15+TGFB1^*^T+CCR5^*^d+IFNAR1^*^G *P_*perm*_* = 0.0056 DRB1^*^15+TGFB1^*^T+CCR5^*^d *P_*perm*_* = 0.013 DRB1^*^15+CCR5^*^d+IFNAR1^*^G *P_*f*_* = 0.014 DRB1^*^15+TGFB1^*^T+IFNAR1^*^G *P_*f*_* = 0.015 Comparative to IFN-beta: CCR5^*^d+IFNAR1^*^G+ DRB1^*^15+TGFB1^*^T *P_*f*_* = 0.00054, *P_*perm*_* = 0.004, OR = 13.2 [3.1–55.4]	–	–
*TCRB* (54)	T-cell receptor beta locus	**rs71878** *P* = 0.015, OR = 6.85 [1.45–31.9]	MHC protein binding peptide antigen binding	T cell costimulation Immune response T cell receptor signaling pathway Regulation of immune response
*CTSS* (54)	Cathepsin S	**rs2275235** *P* = 0.014, OR = 11.59 [1.6–81.9]	Cysteine-type peptidase activity	Toll-like receptor signaling pathway Antigen processing and presentation Adaptive immune response Proteolysis Antigen processing and presentation of exogenous peptide antigen via MHC class II
*EOMES* (53)	Eomesodermin homolog	**rs2371108** T allele, *P_*f*_* = 0.018, OR = 2.00 [1.09–3.66]	Sequence-specific DNA binding transcription factor activity	Mesoderm development regulation of transcription from RNA polymerase II promoter Interferon-gamma production CD8-positive, alpha-beta T cell differentiation involved in immune response Adaptive immune response Cerebral cortex neuron differentiation
*CLEC16A* (53)	Protein CLEC16A	**rs6498169** A allele, *P_*f*_* = 0.025, OR = 2.38 [1.08–5.27]	Protein binding	Catabolic process Cell communication Lysosomal transport organelle organization Regulation of biological process Response to external stimulus Response to stress Regulation of TORC1 signaling
*IL22RA2* (53)	Interleukin-22 receptor subunit alpha-2	**rs202573** GG genotype, *P_*f*_* = 0.0080, OR = 2.08 [1.18–7.41]	Cytokine receptor activity Protein binding Signal transducer activity	Regulation of biological process Response to stimulus Regulation of tyrosine phosphorylation of STAT protein
*PVT1* (53)	Pvt1 oncogene (non-protein coding)	**rs2114358** A allele, *P_*f*_* = 0.0050, OR = 2.77 [1.33–5.77]	–	–
***GWAS -GA***				
*UVRAG* (56)	UV radiation resistance associated	**rs80191572** OR = 0.50 [0.35, 0.75]	Protein binding	Receptor catabolic process Regulation of cytokinesis
*HLA-DQB2* (56)	Histocompatibility complex	**rs28724893** OR = 0.59 [0.47, 0.75]	–	Antigen processing and presentation of peptide or polysaccharide antigen via MHC class IICellular defense response
*MBP* (56)	Myelin basic protein	**rs1789084** OR = 0.54 [0.38, 0.78]	Structural constituent of myelin sheath	Biological regulation cell communication Nervous system development Neurological system process
*ZAK* (*CDCA7*) (56)	Cell division cycle associated 7	**rs139890339** OR = 0.23 [0.10, 0.53]	–	Apoptotic processRegulation of transcription, DNA-templated

### GWAS and Glatiramer Acetate

To date, one GWAS study investigating the research question of pharmacogenomics and GA has been published (56). Patients with extreme phenotypes were included in the analysis, considering a 4-year follow-up period. Genotyping was conducted using Illumina OMNI-5M genome-wide array® covering 4301331 SNPs. Significant associations with treatment response were identified in the following genes: *UVRAG* (rs80191572), *HLA-DQB2* (rs28724893), *MBP* (rs1789084), and *ZAK* (rs139890339). Marginal association with another polymorphism (rs470929) located in the *MBP* gene has previously been reported in the candidate gene study conducted by Grossman et al. (54). The *MBP* gene encodes the autoantigen myelin basic protein, which is attacked by the immune system in MS patients. Furthermore, GA was designed as an MBP mimetic (51). The results from the GWAS study warrant further confirmation in independent studies. Significant results are presented in Table 3.

### Mitoxantrone

Mitoxantrone is synthetic anthracenedione – a cytotoxic agent that inhibits DNA repair via inhibition of topoisomerase II leading to a suppressed proliferation of T cells, B cells, and macrophages, decreased pro-inflammatory cytokine secretion, enhanced suppressor T cell function, and suppressed macrophage-mediated myelin degradation (57). Two studies investigating an association between genetic polymorphisms and mitoxantrone were published to date, providing conflicting results (58, 59). In the first study, authors proposed that SNPs in ABC-transporter genes (*ABCB1* and *ABCG2*) might serve as pharmacogenetic markers associated with clinical response to mitoxantrone therapy in patients with RRMS or SPMS forms of the disease. The second study failed to confirm the association in PPMS form of the disease (59).

### Natalizumab

Natalizumab is a humanized monoclonal antibody that inhibits the migration of lymphocytes via the BBB by inhibiting an adhesive molecule of anti-integrin-α4 (57). Currently, only one pharmacogenetic study in association to treatment response was conducted (60). Authors investigated an association between polymorphisms in *NQO1* and *GSTP1* genes and treatment efficacy. In a combined analysis, it was found that patients who carried the wild-type genotype or only one non-wild polymorphism for either gene have possibly a better clinical outcome after receiving the natalizumab therapy.

### PharmGKB Variants

According to PharmGKB levels of evidence for variant-drug associations, no clinically actionable variants with the level of evidence 1A or 1B exist for MS (October 9^th^, 2018). We identified eleven variant-drug combinations associated with treatment efficacy and with the level of evidence 3, which stands for an annotation based on a single significant (not yet replicated) result or annotation for a variant-drug combination evaluated in multiple studies but lacking clear evidence of an association (61). The PharmGKB variants are presented in Table 4.

**Table 4:** Variant/gene-drug pairs currently listed in PharmGKB database (October 9th, 2018).

**Variant**	**Gene**	**Type**	**Level of evidence**	**Chemicals**
rs12044852	*CD58*	Efficacy	3	Interferon beta-1a, interferon beta-1b
rs4278350	–	Efficacy	3	Interferon beta-1a, interferon beta-1b
rs760316	*FHIT*	Efficacy	3	Interferon beta-1a, interferon beta-1b
rs1448673	–	Efficacy	3	Interferon beta-1a, interferon beta-1b
rs10819043	*GAPVD1*	Efficacy	3	Interferon beta-1a, interferon beta-1b
rs3133084	–	Efficacy	3	Interferon beta-1a, interferon beta-1b
rs10494227	*ZNF697*	Efficacy	3	Interferon beta-1a, interferon beta-1b
rs2291858	*GAPVD1*	Efficacy	3	Interferon beta-1a, interferon beta-1b
rs10760397	*GAPVD1*	Efficacy	3	Interferon beta-1a, interferon beta-1b
HLA-DRB1^*^04:01:01	*HLA-DRB1*	Efficacy	3	Interferon beta-1a
HLA-B^*^15:01:01:01	*HLA-B*	Efficacy	3	Interferon beta-1a

### Neutralizing Antibodies (NAbs)

Part of the unresponsiveness to IFN-beta can also be explained by the development of neutralizing antibodies (NAbs) that reduce the drug efficacy. These develop in up to third of patients, depending on the IFN-beta product administered (62). However, the use of NAbs, as an early pharmacogenetic biomarker is limited because NAbs develop only after 6-24 months from initiation of treatment and patients may even revert to NAbs-negative over time (63). Additionally, NAbs-positivity explains the unresponsiveness to IFN-beta treatment only in a small proportion of patients (64). Nevertheless, it might be useful to include the information on NAbs in the pharmacogenetic studies of MS. Furthermore, the genetic markers that influence the development of NAbs have also been identified in patients with MS (candidate gene studies and GWAS) (48, 65–68).

### Transcriptomic Pharmacogenetic Biomarkers

Although beyond the scope of this paper, several studies indicate that gene expression signatures could prove useful in predicting long-term treatment response in patients with MS (69–71). These studies revealed differences in the expression of genes related to IFN-beta signaling, TLR-4 signaling in monocytes, as well as increased overall molecular response to IFN-beta in non-responders (72). Recently, RNA-sequencing in whole-blood showed that expression of a ribosomal protein S6 was reduced in IFN-beta responders compared to non-responders (73). In another RNA-sequencing study, the different pre-treatment gene expression signature in peripheral blood mononuclear cells (PBMCs) was revealed in MS patients responsive to fingolimod compared to non-responders (74). However, most of the currently proposed transcriptomic biomarkers have only moderate discriminative power and have yet not been validated (75, 76). Additionally, gene expression is more variable than genetic status and largely depends on various environmental factors, drugs co-administered (such as corticosteroids), specific cell populations studied (whole blood, PBMCs, T-cells), and differences in sampling times. Divergent findings can also be explained by the heterogeneity of technical protocols and clinical assessment of treatment response.

## Discussion

In recent years, several actionable pharmacogenomics biomarkers have been identified, comprising many areas of medicine. The implementation of pharmacogenomics in clinical practice has, therefore, a great potential to enable more personalized treatment with several benefits for patients and society. However, despite the increasing number of treatment options available to patients with MS and a high degree of variability in response to these treatments, there is still no reliable pharmacogenomic biomarker that would differentiate between MS-treatment responders and non-responders. Since MS is a chronic progressive disorder, which requires life-long treatment, an early decision for the right therapy may have a high clinical utility for MS patients. By choosing the right treatment for a particular individual early in the disease course, we can slow down the progression of the disease, avoid possible adverse events and improve the efficiency of treatment.

Comprehensive systematic analysis of pharmacogenomic studies showed that the majority of the included studies (87.5%) are limited to candidate genes, mostly hypothesized to be involved in pathways of drug actions. We have observed that candidate gene studies largely lack the replication and confirmation of the results. However, we have identified some genes, the variability of which has been investigated repeatedly, such as *MXA, CCR5, GPC5, IFNAR1, IFNAR2, IRF5, NLRP3* genes, and HLA-region. The results of currently published candidate gene studies were mostly inconsistent, which may in part reflect the various study designs, including the inconsistent approach of defining response to treatment, as well as limited sample sizes with insufficient effect size. Nevertheless, it is evident that biological processes defined by statistically significant genes implicated in IFN-beta response are mostly immune-related and include regulation of interleukins production, positive regulation of regulatory T cell differentiation, negative regulation of cytokine production, type I interferon signaling pathway, mononuclear cell proliferation, cellular response to lipopolysaccharide, cellular response to interferon-gamma, regulation of cytokine-mediated signaling pathway, defense response to virus, leukocyte migration, and regulation of innate immune response.

Despite the proposed distinct immunomodulatory mechanisms of actions of IFN-beta and GA, we have observed that some of the significant associations were identified in the same genes, or in genes involved in the same biological pathways. As an example, it has been found that the polymorphisms of the cathepsin S (*CTSS*) gene are associated with a response to the treatment of both IFN-beta and GA. Cathepsin S has cysteine-type peptidase activity and is involved in several biological processes, including Toll-like receptor signaling pathway, antigen processing and presentation of exogenous peptide antigen via MHC class II, adaptive immune response, and proteolysis, also of human myelin basic protein (MBP) (77). Furthermore, it has been suggested that discriminative allelic variants of the *CCR5, IFNAR1*, and *TGFB1* genes, which are involved in MAPK cascade, defense response, type I IFN signaling pathway, regulatory T cell differentiation, and apoptotic processes, may direct the treatment decision between IFN-beta or GA (6).

In recent years, GWAS studies identified novel candidate genes, which remain to be validated. Moreover, there was no overlap between the top-ranked results of GWAS studies, which suggests that response to existing therapies is influenced by numerous polymorphisms in multiple genes. However, among potential candidate genes identified in GWAS studies of IFN-beta, we detected significant enrichment for genes involved in the glutamate receptor-signaling pathway. Therefore, in the future, more global approaches, such as GWAS or next-generation sequencing (NGS), are required to gain further insight into the pharmacogenomics of MS.

It is important to acknowledge the methodological heterogeneity between the studies included in the present systematic review, such as variability of clinical characterization of the patients, differently defined clinical response, the varying duration of follow-up period among studies, and different genotyping platforms used in GWAS studies. It has previously been reported that the proportion of non-responders varies depending on the definition of treatment response used (78). The clinical criteria for phenotypic classification of patients (responders/non-responders) included: (1) relapse rate (with different thresholds between studies), (2) disease progression, which was most often measured by EDSS score, and (3) changes in MRI activity, such as increase in T2 lesion burden or T1 gadolinium (Gd) enhancing lesions on MRI. The detailed data on the definition of responders/non-responders for each study are presented in Supplementary Table 1. Similarly, the follow-up period ranged from six months (in one study) to 1 year in one study, two years in the majority of the included studies and to four years in two recent studies.

More independent studies investigating the association between already proposed polymorphisms in genes, such as *GRHL3, NINJ2, TBXAS1, GRM3, GRIK2*, and *SLC9A9* and treatment response are warranted to establish reliable and accurate pharmacogenomics predictors. Future studies need to include a larger number of subjects of various ethnicities. It is also crucial to use uniform and precise definitions of treatment response, standardized duration of the follow-up period, and comprehensive clinical characterization of patients.

Also, the GWAS studies are limited to common variants. Of note, rare variants contribute a major part of pharmacogenetic variability (79). In recent years, many important advances in sequencing technologies have been achieved that will in future enable a more comprehensive picture of pharmacogenetic variability in MS patients. Further studies should also consider rare variants obtained by NGS technologies, such as exome or genome sequencing data. To the best of our knowledge, no study investigating the rare variation in exome or genome sequencing data of MS patients in association to treatment response has been published to date.

Furthermore, we suppose that the phenotype of the response to an immunomodulatory pleiotropic therapy, such as IFN-beta and GA, is a sum of numerous contributing genetic factors that were not sufficiently simultaneously and combinatorially assessed by current study designs and methodologies. More studies investigating cumulative effects of polymorphisms in multiple genes (additive effects or epistatic interactions), such as studies of Kulakova et al. (34, 53), are needed to gain a more comprehensive insight into genetic variability in association to the efficacy of treatment.

Another important aspect of future pharmacogenomics, especially for the interpretation of rare variants, are publicly available and easily updatable databases of pharmacogenomic variation, such as PharmGKB, CPIC, ClinVar, as well as population-specific databases. Further standardized dosing recommendations and guidelines based on the patient's genomic test results are required, ideally integrated with demographic, phenotypic and clinical data.

Furthermore, most of the collected studies (94%) were conducted on patients treated with IFN-beta or GA. Lack of pharmacogenomic studies conducted on drugs approved in recent years, such as dimethyl fumarate (Tecidifera®), teriflunomide (Aubagio®), and fingolimod (Gilenya®) limits the implementation of personalized medicine into clinical practice. An increasing number of new treatment options will in future enable more personalized treatment approaches; however, many genome-wide studies carried out on large sample sizes and in different populations are needed to reach reliable pharmacogenomics biomarkers for implementation into daily clinical practice.

In conclusion, current literature data suggests that genetic variability can significantly contribute to the response to treatment in patients with MS. In the future, it is necessary to systematically evaluate the polymorphisms that were previously proposed to influence the response to treatment as well as assess the importance of rare variants and their effects on the treatment of MS. Additional studies and larger ethnically homogenous cohorts are necessary to provide new insides and optimized use of MS drugs. More combinatorial study designs are needed to assess the effect of several combinations of polymorphisms in various genes simultaneously to provide more relevant information for the clinical use of pharmacogenomics. Studies investigating the pharmacogenomics of newer medicines for MS are also necessary, using the clear and uniform criteria of defining treatment response. We believe that all of the above, along with the rapid development of new medications and advances in genomic technologies, will in future enable a more personalized approach to MS treatment.

## Author Contributions

BP, SR, and KH contributed conception and design of the study; KH and SR searched the database for potentially eligible articles and extracted the data. KH wrote the original draft. SR and BP contributed to manuscript revision. All authors reviewed the final version of the manuscript prior to submission for publication.

### Conflict of Interest Statement

The authors declare that the research was conducted in the absence of any commercial or financial relationships that could be construed as a potential conflict of interest.

## Footnotes

^1^http://www.prisma-statement.org/.

## Abbreviations

ARR: annualized relapse rate
BBB: blood-brain barrier
CNS: central nervous system
CPIC: Clinical Pharmacogenetics Implementation Consortium
EDSS: expanded disability status scale
GA: glatiramer acetate
GO: gene ontology
GWAS: genome-wide association study
IFN-beta: interferon beta
MHC: major histocompatibility complex
MRI: magnetic resonance imaging
MS: multiple sclerosis
Nabs: neutralizing antibodies
PBMCs: peripheral blood mononuclear cells
PharmGKB: The Pharmacogenomics Knowledgebase
PPMS: primary progressive multiple sclerosis
PRISMA: Preferred Reporting Items for Systematic Reviews and Meta-Analyses
RRMS: relapsing-remitting multiple sclerosis
SPMS: secondary progressive MS.
